# Decoding the mystery between hyperuricemia and atrial fibrillation: new causal links through mediating proteomics

**DOI:** 10.3389/fendo.2025.1429465

**Published:** 2025-05-21

**Authors:** Meiwei Zhang, Lei Shi, Cong Qin, Mengling Peng, Zhiguo Zhang

**Affiliations:** Department of Cardiovascular Diseases, The First Hospital of Jilin University, Changchun, Jilin, China

**Keywords:** atrial fibrillation, hyperuricemia, mediation mendelian randomization, proteomics, mediation analysis

## Abstract

**Background:**

Atrial fibrillation (AF), the most common cardiac arrhythmia, is associated with high incidence and mortality rates. Recent studies have confirmed a close correlation between hyperuricemia and the onset of AF, though the mechanisms remain unclear. Consequently, this study employs Mendelian randomization based on proteomics and mediation analysis to investigate the potential mechanisms by which hyperuricemia induces AF.

**Methods:**

A two-step mediation MR analysis was conducted to determine whether plasma proteins mediate atrial fibrillation induced by serum urate. The Reactome database was subsequently utilized to analyze the list of significant mediating plasma proteins to identify enriched pathways.

**Results:**

Mediation Mendelian randomization analysis suggested that hyperuricemia may promote the development of atrial fibrillation (AF) through 17 plasma proteins, includeing hepatocyte nuclear factor 4-alpha (HNF4α), identified as key mediators. Subsequent enrichment analysis of these proteins revealed 9 metabolic or signaling pathways potentially involved in this pathological process. Central mediator proteins such as HNF4α appear to drive AF through metabolic and inflammatory pathways.

**Conclusion:**

There is a close correlation between hyperuricemia and the onset of atrial fibrillation.

## 1 Introduction

Atrial fibrillation (AF), as the most common cardiac arrhythmia, has seen an annual increase in global incidence and prevalence. According to data from the Framingham Heart Study (FHS), the prevalence of AF has tripled over the fifty years from 1958 to 2007 (1). From 2010 to 2060, the number of adults aged 55 and over with AF in the European Union is expected to more than double (2).The primary risk factors for AF include advanced age, hypertension, obesity, diabetes, heart failure, valvular heart disease, and myocardial infarction, among others (3).Besides significantly impacting patients’ quality of life, AF also substantially increases the risk of morbidity and mortality from conditions such as heart failure, thromboembolism, and stroke (4).Current management of atrial fibrillation primarily involves controlling heart rate and rhythm to improve symptoms and prevent strokes, while also managing comorbidities and lifestyle factors. Although ablation and risk management strategies for AF have yielded some improvement, the prevalence of AF continues to rise. Given the complexity, progressive nature, and limited detectability of atrial fibrillation, coupled with a high incidence of treatment ineffectiveness, the prevention and treatment of atrial fibrillation face increasing challenges (5, 6).Hyperuricemia is a metabolic syndrome caused by a disorder in purine metabolism, with uric acid being the final product of purine catabolism in the body. Under physiological conditions, the synthesis and excretion of uric acid are balanced. Once this balance is disrupted, hyperuricemia will ensues. In recent years, extensive clinical research has confirmed the association between hyperuricemia and atrial fibrillation (7–11). Numerous studies indicate that hyperuricemia promotes the development of cardiovascular diseases through mechanisms such as the modulation of inflammatory responses (12–14), oxidative stress (15), insulin resistance (16), endothelial dysfunction (17), and endoplasmic reticulum stress (18, 19). Although the relationship is widely recognized, the mechanisms by which hyperuricemia induces or sustains atrial fibrillation remain incompletely elucidated. The level of evidence from Mendelian Randomization (MR) studies lies between that of randomized controlled trials (RCTs) and observational studies (20). It utilizes lineage-specific genetic variants as instrumental variables (IVs) to explore the causal relationships between exposure phenotypes and outcome phenotypes (21). According to Mendel’s laws, alleles are randomly distributed from parents to offspring, and the genotype precedes exposure temporally (22).

Consequently, MR studies can minimize confounding factors to the greatest extent and eliminate reverse causation. This study aims to investigate the underlying mechanisms by which hyperuricemia leads to AF, using proteomic Mendelian randomization and mediation analysis methods. It identifies the link between purine metabolism disorders and the onset of AF, thereby determining potential therapeutic targets for AF.

## 2 Methods

### 2.1 Data source

As shown in **Table 1**, the GWAS summary data related to serum urate levels were published by Köttgen et al. in 2013 (23). This research measured serum urate levels and performed whole-genome sequencing on 110,347 individuals from 48 studies, followed by GWAS and meta-analysis. Prior to all meta-analyses, monomorphic SNPs were excluded. If the genomic inflation factor of the study exceeded 1, all study-specific results were corrected using the genomic inflation factor, calculated by dividing the median of the observed GWAS chi-square distribution by the median of the expected chi-square distribution under the null hypothesis of no association. GWAS data associated with atrial fibrillation were published by Nielsen et al. in 2018 (24). This study conducted a genome-wide association analysis on over 1,000,000 individuals, including 60,620 cases of atrial fibrillation and 970,216 controls. Atrial fibrillation patients were identified based on ICD-10 code I48 and ICD-9 code 427.3 in electronic medical records. Plasma proteome-based GWAS data were released by Sun et al. in 2018. The study, conducted from mid-2012 to mid-2014, recruited donors aged 18 and above at 25 centers of the National Health Service Blood and Transplant (NHSBT) in England, excluding those with a history of major diseases such as myocardial infarction, stroke, cancer, HIV, and hepatitis B or C, or recent illness or infection. A multiplex aptamer-based approach (SOMAscan assay, with standardized and normalized data) was utilized to measure the relative concentrations of 3,622 plasma proteins or protein complexes, using 4,034 modified aptamers (25). The aforementioned data have each passed an ethical review and include European populations of both genders, effectively mitigating biases introduced by population stratification. Details regarding cohort recruitment and the ethical approval of the original studies can be found in **Supplementary Files 1, 2**.

**Table 1:** Information on the included data sets.

Trait	Case	Sample size	Year	Author	Gender	Population	NSNP
Atrial fibrillation	60620	1030836	2018	Nielsen JB	Males and Females	European	33519037
Urate	110347	110347	2013	GUGC	Males and Females	European	2450548
3282 plasma proteome	3301	3301	2018	Sun BB	Males and Females	European	10534735

### 2.2 Selection of instrumental variables

Extraction of valid instrumental variables is key to conducting MR analysis; these variables must meet the relevance, independence, and exclusion criteria of the MR assumptions. Initially, we extracted exposure-related SNPs at a genome-wide significance level (*P*-value less than 5 × 10^^-8^). In the mediation MR analysis, due to varying associations between different plasma proteins and genetic variants, extracting too many SNPs would increase heterogeneity, while extracting too few would result in insufficient explained variance. Therefore, a second threshold was established (*P*-value less than 1 × 10^^-5^). SNPs exhibiting linkage disequilibrium (LD) were removed based on an r^^2^ < 0.001 and a window size > 10,000 kb. Data on SNPs associated with outcomes are extracted based on SNP identifiers in the outcome datasets. Ambiguous SNPs and palindromic SNPs were eliminated in this process, aligning the SNP data from both datasets. The F statistic was calculated for each SNP (26). The F statistic, an intermediate measure in analysis of variance, quantifies the associative strength of the instrumental variable SNPs with risk factors. SNPs with an F statistic below 10 were considered weak instrumental variables and were excluded. The MR-PRESSO test was conducted to identify and exclude potential pleiotropic SNPs. The MR-Stiger test was applied to examine the directional causality of each SNP, excluding those with incorrect directions (27). Finally, the PhenoScanner test was used to determine if any SNPs were associated with confounding factors, removing those that potentially violated the independence assumption (28). Following the aforementioned filtering, the remaining SNPs were deemed compliant with the three major MR assumptions and considered valid instrumental variables.

### 2.3 Mendelian randomization analysis

Classical Mendelian Randomization requires the use of Inverse Variance Weighting (IVW). To evaluate the robustness of the results, we also employed MR Egger regression, Weighted Median (WM), Mode-based estimation, and MR Robust Adjusted Profile Score (MR-raps) to comprehensively assess potential biases in the study findings. The IVW method consolidates causal estimates of individual SNPs using a variance inverse-weighted form of the Wald ratio (29). The Wald ratio estimates measure the impact of a single SNP on the outcome relative to its effect on the risk factor, assuming that all associations conform to a log-linear relationship (30). MR-Egger regression, a valuable tool in MR analysis, is used to establish a weighted linear regression between the outcome and exposure coefficients (31). MR-Egger regression, similar to the IVW method, allows for the assessment of horizontal pleiotropy through the significance level of its intercept term. The MR-Egger method is based on the No Measurement Error (NOME) assumption. We also calculated the I^^2^ statistic to quantify the extent to which MR-Egger violates the NOME assumption. When I^^2^ is less than 90%, the results should be adjusted (31, 32). When multiple variants are ineffective, the results of the aforementioned method may lack robustness, in which case the WM method and the weighted mode demonstrate greater robustness. The WM method calculates normalized inverse-variance weights for each genetic variant, then combines these weights to generate an estimate. Importantly, as long as at least 50% of the weights used in the analysis come from valid instrumental variables, the WM method can provide reliable estimates of causal effects. Even in the presence of some invalid instrumental variables, the WM method can accurately estimate causal relationships, enhancing precision. The weighted mode remains robust even with a greater number of invalid instrumental variables. Furthermore, this study employs the newly developed MR-RAPS technique, which directly simulates the pleiotropic effects of genetic variants using a random-effects distribution. Compared to traditional Mendelian randomization techniques, this novel strategy offers enhanced robustness. When the *P*-value is less than 0.05, the final results are statistically significant.

### 2.4 Sensitivity analysis

Pleiotropy encompasses both horizontal and vertical dimensions; typically, vertical pleiotropy does not compromise the reliability of the conclusions; however, horizontal pleiotropy should be eliminated. The primary method employed for estimating the magnitude of horizontal pleiotropy is the MR-Egger approach. If the *P*-value of the MR-Egger intercept is less than 0.05, the instrumental variables are considered to be significantly affected by horizontal pleiotropy, rendering the results unreliable. When pleiotropy is present, MR-Egger regression is utilized as the principal analytical method.

In MR analysis, even if all SNPs are valid instrumental variables, they may exhibit heterogeneity. The presence of substantial heterogeneity can compromise the reliability of the findings; thus, heterogeneity tests are conducted to enhance the credibility of the results. The IVW method is used to calculate heterogeneity among SNPs, assessed with Cochran’s Q test. A *P*-value less than 0.05 indicates heterogeneity; in such cases, an IVW random effects model is applied alongside the weighted median method. If no heterogeneity is present, the IVW random effects model remains the main analytical approach (33). Additionally, as per convention, the leave-one-out method is employed, and a funnel plot is constructed. A comprehensive MR-Steiger test was conducted to verify the correct overall causal direction. Finally, we calculated the statistical power to ascertain the reliability of negative results (34).

### 2.5 Proteomic mediation analysis and enrichment analysis

Two-step mediation MR analyses using GWAS summary data were conducted to determine whether plasma proteins are intermediary factors in serum urate-induced atrial fibrillation. The first step involved a two-sample MR analysis between serum urate and the plasma proteome, followed by a second two-sample MR analysis between the plasma proteome and atrial fibrillation. Proteins significant in both analyses exhibited partial mediation effects, those only significant in the two-step mediation analyses showed complete mediation effects, and proteins not consistently significant displayed no mediation effects. Indirect effects were calculated using the formula β1*β2, and direct effects were determined by subtracting indirect effects from the total effects. The Reactome knowledgebase (35) was utilized to analyze the list of mediating plasma proteins to understand enriched pathways. Reactome is a peer-reviewed database of human biological pathways and reactions. Overrepresentation analysis was conducted to determine whether specific Reactome pathways were enriched in the gene list, generating probability scores and significance *P*-values.

### 2.6 Statistical software and visualization

For the visualizations in the conclusion section, this study generated scatter plots for each SNP, illustrating the relationship with exposure factors and outcome effects, accompanied by regression curves to present causal estimates. A significance heatmap for the MR analysis was created to display the results. Funnel plots were utilized to assess potential directional effects and pleiotropy, as well as to examine the distribution of data. The final causal estimates were used to create forest plots, which displayed the results for each SNP and the overall MR analysis outcomes. All statistical analyses in this study were conducted using R (version 4.2.3) and the R packages “TwoSample MR”, “MR-PRESSO”, and “mr.raps”.

## 3 Results

### 3.1 Selection of instrumental variables

In the MR analysis, initially 27 SNPs associated with exposure were screened, and no weak instrumental variables were identified; no SNPs were excluded from the outcome database due to missing data. 2 SNPs were identified as ambiguous or palindromic, and 2 SNPs were excluded after screening with Phenoscanner due to their association with confounding factors. The MR-PRESSO test identified 2 SNPs with horizontal pleiotropy; the MR-Steiger test found no SNPs with incorrect causal direction. No SNPs directly related to the outcome were identified; ultimately, 21 eligible SNPs were included in the study. In the mediation MR analysis, initially, 193,706 exposure-related SNPs were screened without detecting any weak instruments; 3,171 SNPs were excluded from the outcome database due to missing data. A total of 21,886 ambiguous and palindromic SNPs were removed during the dataset consolidation. The MR-PRESSO test detected no SNPs with horizontal pleiotropy; the MR-Steiger test revealed no SNPs with erroneous causal directions. Following Bonferroni correction, 12,469 SNPs directly related to the outcome were removed, leaving 156,180 eligible SNPs included in the study.

### 3.2 Mendelian randomization analysis

**Figure 1:**
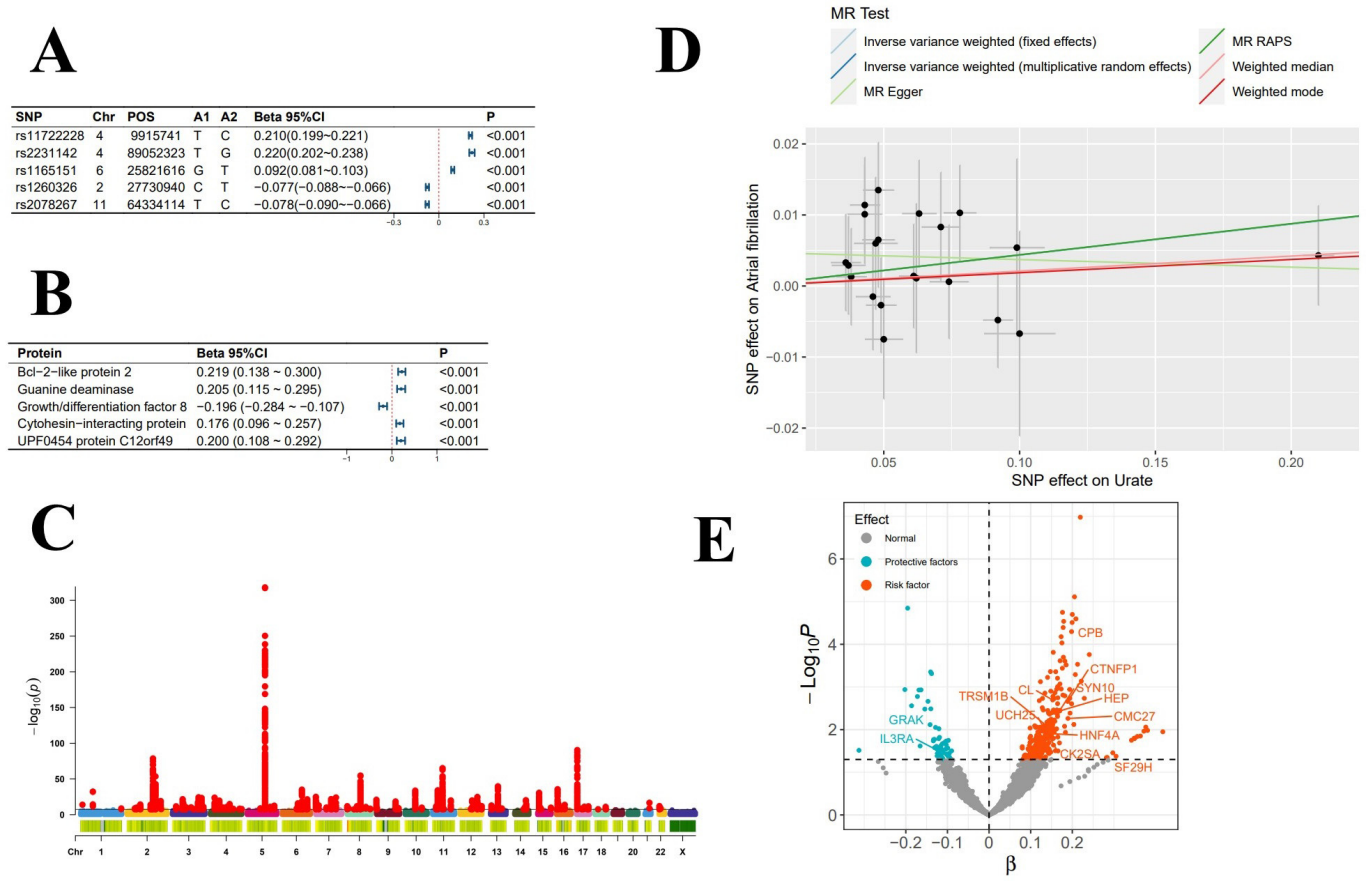
Exposure-Related Analysis Graphs. **(A)** Top 5 single nucleotide polymorphisms (SNPs) most significantly associated with serum urate levels; **(B)** Top 5 plasma proteins most significantly impacted by serum urate levels; **(C)** Manhattan plot of genome-wide association study summary data for serum urate levels; **(D)** Scatter plot and regression curve from Mendelian randomization exploring the causal relationship between serum urate levels and atrial fibrillation; **(E)** Scatter plot of the significance levels of serum urate effects on plasma proteins, with specified proteins indicating mediation.

In the Mendelian Randomization analysis, an increase in serum urate levels was found to promote the onset of atrial fibrillation (OR (95% CI): 1.045 (1.007 ~ 1.083), *P*=0.019, see **Figure 1D**). There was no evidence of pleiotropy or heterogeneity in these results.

**Figure 2:**
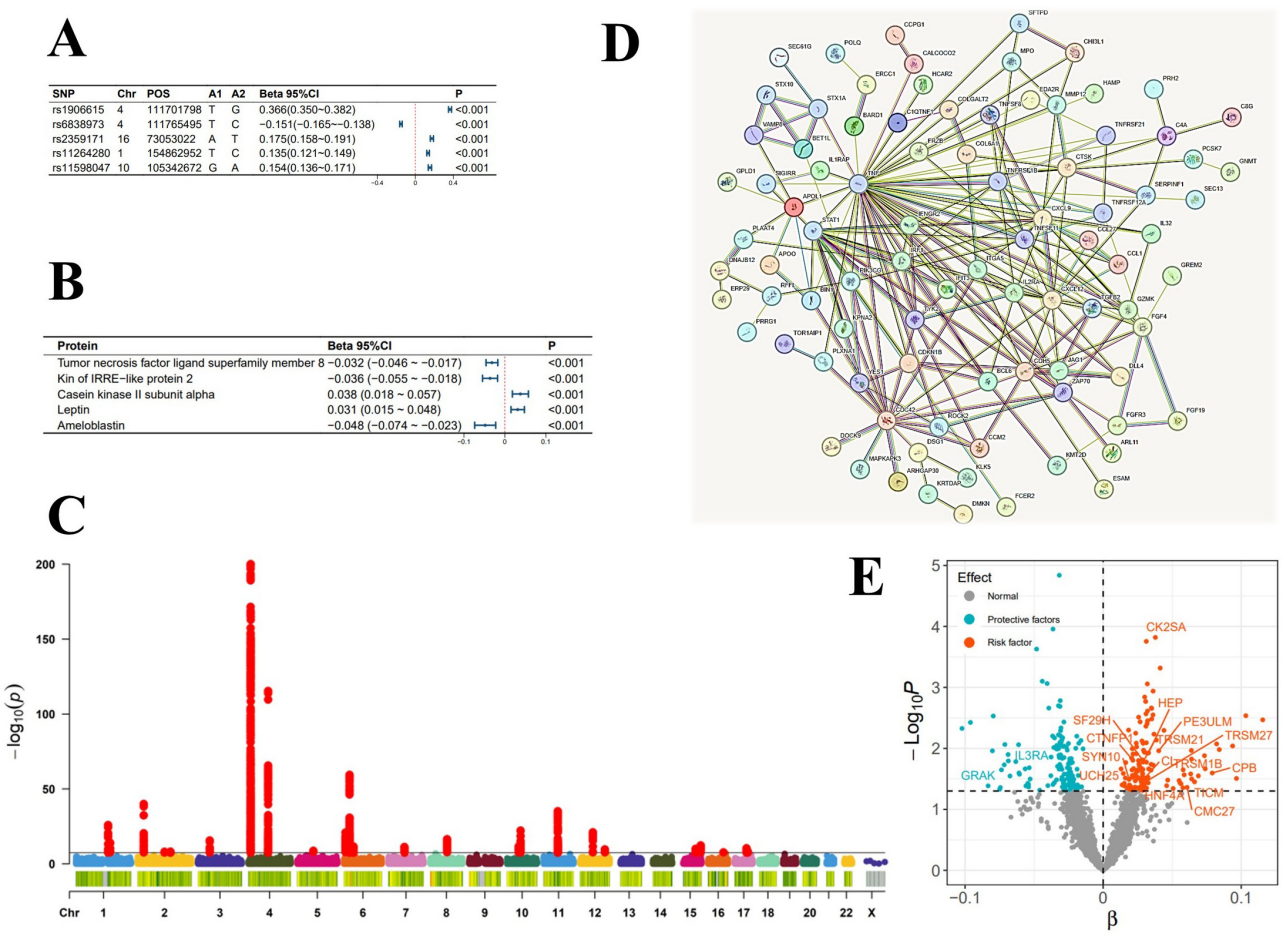
Analysis Graphs Related to the Incidence of Atrial Fibrillation. **(A)** Top 5 SNPs most significantly influencing the incidence of atrial fibrillation; **(B)** Top 5 plasma proteins most significantly associated with the incidence of atrial fibrillation; **(C)** Manhattan plot of genome-wide association study summary data for atrial fibrillation; **(D)** Diagram of plasma protein interactions affecting the incidence of atrial fibrillation; **(E)** Scatter plot of the significance levels of plasma protein effects on the incidence of atrial fibrillation, with specified proteins indicating mediation.

**Figure 3:**
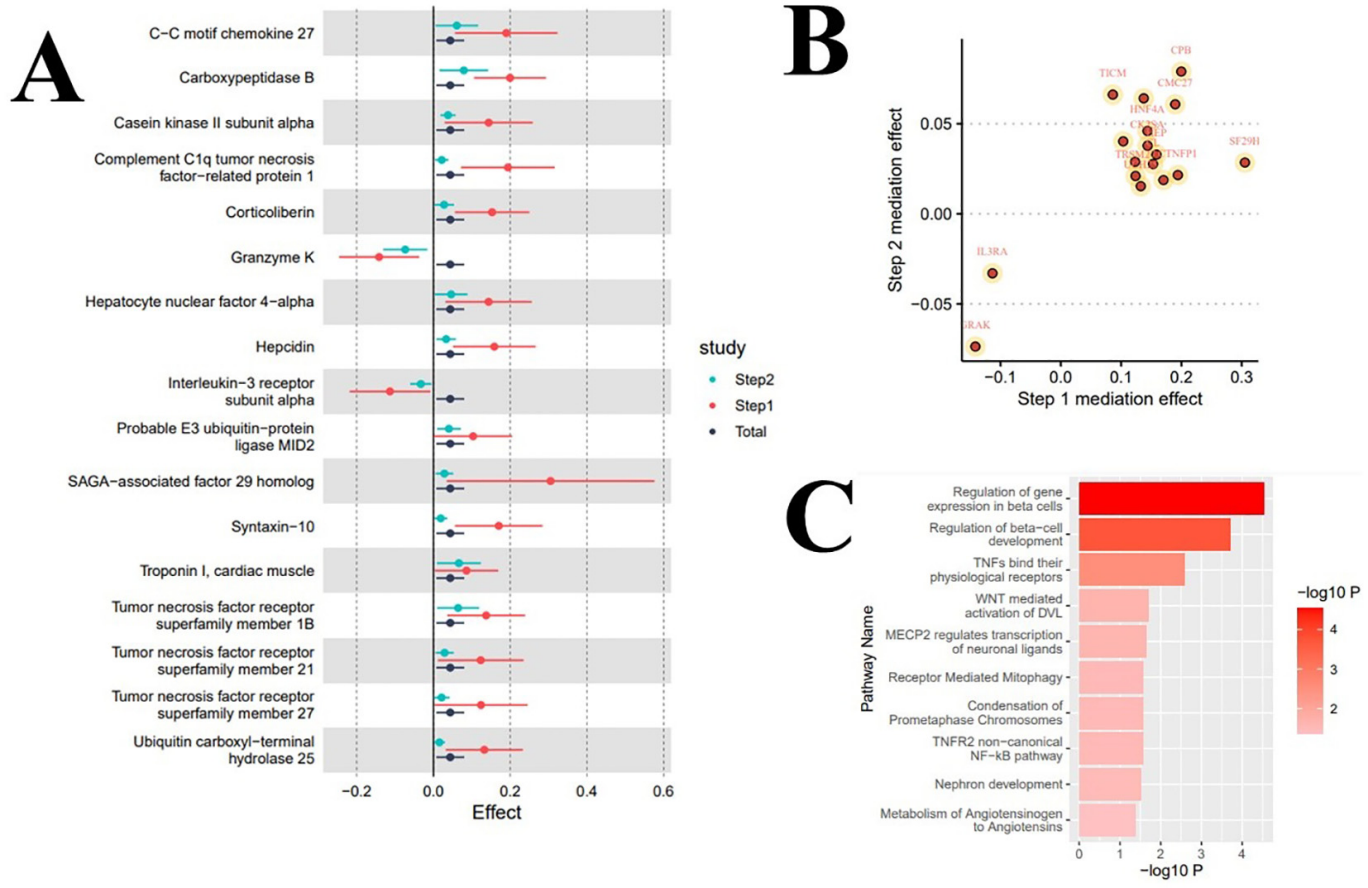
Results of mediation analysis and enrichment analysis. **(A)** Forest plot of total effect and two-step mediation effects; **(B)** Scatter plot of two-step effects of mediating proteins; **(C)** Mediating pathways of metabolic or signal transduction related to serum urate levels in the onset of atrial fibrillation.

In the mediation MR analysis, we initially discovered that serum urate exerted a causal effect on 348 proteins (**Figures 1B, E**), and 269 proteins causally influenced the onset of atrial fibrillation (**Figures 2B, E**). Subsequently, a protein-protein interaction analysis was conducted on plasma proteins that induce atrial fibrillation (**Figure 2D**). Ultimately, we identified 17 plasma proteins with mediating effects, including Hepatocyte nuclear factor 4-alpha (**Table 2**, **Figures 3A, B**), which mediated the promotion of atrial fibrillation by serum urate, with mediation effect ratios ranging from 0.05% to 0.36%.

**Table 2:** Significant results of two-step mediation analysis.

Mediator	X-Y		X-M		M-Y		Mediating direction	Mediating effect	Mediating ratio
	OR 95%CI	*P*	OR 95%CI	*P*	OR 95%CI	*P*			
Complement C1q tumor necrosis factor-related protein 1	1.045(1.007~1.083)	0.019	1.214(1.075~1.372)	0.002	1.022(1.004~1.040)	0.002	TRUE	Partial	0.10%
SAGA-associated factor 29 homolog	1.045(1.007~1.083)	0.019	1.357(1.035~1.779)	0.042	1.029(1.005~1.053)	0.042	TRUE	Partial	0.20%
C-C motif chemokine 27	1.045(1.007~1.083)	0.019	1.209(1.057~1.382)	0.005	1.063(1.005~1.124)	0.005	TRUE	Partial	0.26%
Carboxypeptidase B	1.045(1.007~1.083)	0.019	1.221(1.112~1.341)	<0.001	1.082(1.016~1.153)	<0.001	TRUE	Partial	0.36%
Corticoliberin	1.045(1.007~1.083)	0.019	1.165(1.057~1.284)	0.002	1.028(1.002~1.055)	0.002	TRUE	Partial	0.10%
Casein kinase II subunit alpha	1.045(1.007~1.083)	0.019	1.155(1.029~1.295)	0.014	1.039(1.018~1.059)	0.014	TRUE	Partial	0.12%
Tumor necrosis factor receptor superfamily member 27	1.045(1.007~1.083)	0.019	1.132(1.002~1.278)	0.046	1.021(1.000~1.043)	0.046	TRUE	Partial	0.06%
Granzyme K	1.045(1.007~1.083)	0.019	0.868(0.782~0.963)	0.008	0.929(0.877~0.984)	0.008	TRUE	Partial	0.24%
Hepcidin	1.045(1.007~1.083)	0.019	1.172(1.052~1.305)	0.004	1.033(1.007~1.060)	0.004	TRUE	Partial	0.12%
Hepatocyte nuclear factor 4-alpha	1.045(1.007~1.083)	0.019	1.154(1.032~1.292)	0.012	1.047(1.004~1.092)	0.012	TRUE	Partial	0.15%
Interleukin-3 receptor subunit alpha	1.045(1.007~1.083)	0.019	0.893(0.804~0.992)	0.035	0.968(0.941~0.995)	0.035	TRUE	Partial	0.09%
Probable E3 ubiquitin-protein ligase MID2	1.045(1.007~1.083)	0.019	1.109(1.001~1.228)	0.047	1.041(1.009~1.074)	0.047	TRUE	Partial	0.10%
Syntaxin-10	1.045(1.007~1.083)	0.019	1.185(1.057~1.329)	0.004	1.019(1.002~1.037)	0.004	TRUE	Partial	0.07%
Tumor necrosis factor receptor superfamily member 1B	1.045(1.007~1.083)	0.019	1.147(1.037~1.270)	0.008	1.066(1.009~1.127)	0.008	TRUE	Partial	0.20%
Tumor necrosis factor receptor superfamily member 21	1.045(1.007~1.083)	0.019	1.131(1.011~1.265)	0.031	1.029(1.005~1.054)	0.031	TRUE	Partial	0.08%
Troponin I, cardiac muscle	1.045(1.007~1.083)	0.019	1.090(1.003~1.184)	0.042	1.068(1.009~1.131)	0.042	TRUE	Partial	0.13%
Ubiquitin carboxyl-terminal hydrolase 25	1.045(1.007~1.083)	0.019	1.142(1.033~1.262)	0.010	1.015(1.001~1.030)	0.010	TRUE	Partial	0.05%

OR, Odds ratio; CI, Confidence interval; X-Y, Total effect; X-M, step 1 mediating effect; M-Y, step 2 mediating effect.

### 3.3 Enrichment analysis

In the enrichment analysis, we identified 10 significant pathways mediating the increased levels of serum urate in promoting the onset of atrial fibrillation, with evidence remaining for 2 significant pathways after correction for the false discovery rate. Specifically, the pathways ‘Regulation of gene expression in beta cells’ (Reactions: 1/12; Entities: 3/23; Adj. *P*=0.003) and ‘Regulation of beta-cell development’ (Reactions: 1/26; Entities: 3/44; Adj. *P*=0.009) significantly mediated the influence of serum urate on the onset of atrial fibrillation; eight signal transduction pathways including ‘TNFs bind their physiological receptors’ potentially mediated this effect. **Table 3** and **Figure 3C** display the enrichment information for the mediating pathways, with detailed data available in the **Supplementary Materials**.

**Table 3:** Activated mediating metabolism or signal transduction pathways.

Pathway name	Reactions found	Reactions ratio	Entities found	Entities ratio	*P*Value	FDR	Mapped proteins
Regulation of gene expression in beta cells	1/12	0.0008	3/23	0.0020	<0.001	0.003	Hepatocyte nuclear factor 4-alpha
Regulation of beta-cell development	1/26	0.0018	3/44	0.0037	<0.001	0.009	Hepatocyte nuclear factor 4-alpha
TNFs bind their physiological receptors	2/13	0.0009	2/30	0.0025	0.003	0.083	Tumor necrosis factor receptor superfamily member 27;Tumor necrosis factor receptor superfamily member 1B
WNT mediated activation of DVL	1/4	0.0003	1/8	0.0007	0.020	0.301	Casein kinase II subunit alpha
MECP2 regulates transcription of neuronal ligands	1/8	0.0006	1/9	0.0008	0.023	0.301	Corticoliberin
Condensation of Prometaphase Chromosomes	1/4	0.0003	1/11	0.0009	0.028	0.301	Casein kinase II subunit alpha
Receptor Mediated Mitophagy	1/7	0.0005	1/11	0.0009	0.028	0.301	Casein kinase II subunit alpha
TNFR2 non-canonical NF-kB pathway	9/44	0.0030	2/102	0.0087	0.028	0.301	Tumor necrosis factor receptor superfamily member 27;Tumor necrosis factor receptor superfamily member 1B
Nephron development	1/17	0.0012	1/12	0.0010	0.030	0.301	Hepatocyte nuclear factor 4-alpha
Metabolism of Angiotensinogen to Angiotensins	1/18	0.0012	1/17	0.0014	0.042	0.337	Carboxypeptidase B

## 4 Discussion

Atrial fibrillation, as the most common cardiac arrhythmia, severely impacts the quality of life of patients and significantly increases the risk of developing conditions such as heart failure, thromboembolism, and stroke, thereby elevating mortality rates. Although numerous studies have confirmed the association between hyperuricemia and atrial fibrillation (9–11, 36), the precise mechanisms by which hyperuricemia induces atrial fibrillation remain unclear. It is widely believed that atrial remodeling is a prerequisite for the onset of atrial fibrillation, with oxidative stress and inflammation being the most critical mechanisms (37). Mediators of inflammation can alter atrial electrophysiology and structural matrix, as well as regulate calcium homeostasis and connexins, thereby promoting the onset of atrial fibrillation (38). Studies have demonstrated that activation of the NLRP3 inflammasome in atrial myocytes is a potential pathogenic mechanism for atrial fibrillation (12). In a hyperuricemic state, activation of the NLRP3 inflammasome can also promote inflammation, closely resembling the pathogenic mechanism of atrial fibrillation. Additionally, uric acid (UA)-induced upregulation of Kv1.5 expression may represent a novel mechanism for the induction of atrial fibrillation: UA enhances Kv1.5 protein expression by activating the ERK pathway and promoting the expression of Heat Shock Protein 70 (Hsp70) in mouse atrial myocytes (HL-1 cells), thereby increasing the ultra-rapid delayed rectifier K+ current and shortening the duration of action potentials (39, 40). Oxidative stress is also a significant cause of atrial fibrillation in hyperuricemia. In a hyperuricemic state, enhanced oxidative stress responses lead to excessive production of reactive oxygen species (ROS), which by affecting ion channels and the propagation of action potentials, promote atrial fibrillation (41): Hydrogen peroxide induces triggered activity by enhancing late Na+ currents, leading to early afterdepolarizations (EAD) and delayed afterdepolarizations (DAD); Additionally, excessive ROS upregulates L-type Ca2+ channels, altering intracellular calcium homeostasis to promote EADs (41), activates calcium/calmodulin-dependent protein kinase II (CaMKII), increases the opening probability of RYR2 (calcium release channel 2), leading to calcium overload and the formation of multiple waves, thereby inducing atrial fibrillation (42).

Furthermore, oxidative stress can induce atrial fibrillation through atrial structural remodeling, where hydroxyl radicals (OH-) alter the structure and function of myofibrillar proteins, contributing to myocardial damage and the onset of atrial fibrillation (43).

In this study, employing proteomic Mendelian randomization and a two-step mediation analysis, we explored the underlying mechanisms by which hyperuricemia induces atrial fibrillation. We identified 17 proteins playing significant roles in this process, with enrichment analysis revealing that abnormalities in HNF4α leading to beta-cell impairment in the pancreas are critical in the onset of AF driven by hyperuricemia. Hepatocyte nuclear factor 4 alpha (HNF4α) is a nuclear transcription factor (TF) predominantly expressed in the liver, intestines, kidneys, and pancreas (44). It plays a crucial role in transcriptional regulation in hepatocytes and pancreatic cells and is closely associated with carbohydrate and lipid metabolism, and inflammatory responses (45–47). Hyperuricemia may affect transcription factors’ DNA-binding capacity and activity through oxidative stress responses. Additionally, the release of inflammatory cytokines can interfere with the expression and activity of HNF4α. Furthermore, signal transduction activated by inflammatory pathways may indirectly influence the expression or functionality of HNF4α via pathways (48). Abnormal activity and status of HNF4a affect its regulation of downstream genes, ultimately leading to developmental and functional dysfunctions in pancreatic β-cells. Initially, HNF4a plays a crucial role in pancreatic β-cells, influencing the synthesis and secretion of insulin, as well as other functions related to glucose metabolism. Abnormalities in HNF4a are commonly associated with a high incidence of maturity-onset diabetes of the young (MODY), with MODY1 patients exhibiting defects in insulin secretion stimulated by glucose and arginine, indicating a progressive loss of pancreatic β-cell function (49). This also demonstrates that abnormalities in HNF4a lead to the loss of pancreatic β-cell function, exacerbating disorders in glucose metabolism, resulting in persistent hyperglycemia, and further intensifying oxidative stress and inflammatory responses within the body. Moreover, the absence of HNF4α in the liver leads to the dysregulation of multiple genes involved in lipid metabolism, resulting in severe lipid disorders, further intensifying oxidative stress and inflammatory responses in the body (46). Furthermore, increasing evidence suggests a strong link between transcription factor modifications mediated by HNF4α and inflammation/oxidative stress. For instance, the *PARA* gene is one of the known direct target genes of HNF4α (50). Upon activation by HNF4a, the *PPAR* gene not only exerts its anti-inflammatory activity by stimulating the catabolic metabolism of pro-inflammatory arachidonic acid (51), but also inhibits the activation of inflammatory response genes (such as cytokines, metalloproteinases, and acute-phase proteins) through the suppression of the NF-κB signaling pathway, thereby reducing the production of inflammatory mediators (52). Therefore, aberrant expression of HNF4a contributes to the onset of atrial fibrillation by exacerbating metabolic dysregulation, oxidative stress, and inflammatory responses. Besides its effects on pancreatic β-cells, Hou et al. discovered that Peli1-mediated ubiquitination of HNF4α leads to impaired fatty acid oxidation (FAO), a notable metabolic remodeling found in pathological cardiac hypertrophy (53). Such structural changes may also be a causative factor in atrial fibrillation induced by HUA. At the metabolic level, HNF4α maintains myocardial energy homeostasis by regulating genes involved in fatty acid oxidation (FAO); its dysfunction may lead to electrical remodeling (e.g., impaired calcium handling) and structural remodeling (e.g., fibrosis) (53). In addition, HNF4α acts as a transcriptional activator of TGF-β1, promoting atrial matrix remodeling by upregulating TGF-β1 expression, which in turn suppresses the miR-29 family and facilitates CDK2-mediated fibroblast proliferation (54). Moreover, the protein stability of HNF4α is modulated by ubiquitination—such as Peli1-mediated modification at K307/K309 residues—and by stress-responsive signaling pathways including AMPK and JNK2. Aberrant degradation of HNF4α may contribute to AF pathogenesis by exacerbating metabolic imbalance and autophagy overactivation (55).

In addition to the aforementioned pathways, we have identified potential activations of 17 plasma proteins along eight metabolic and signaling pathways: TNFs binding their physiological receptors, WNT-mediated activation of DVL, MECP2 regulation of neuronal ligands transcription, Condensation of Prometaphase Chromosomes, Receptor-Mediated Mitophagy, TNFR2 non-canonical NF-kB pathway, Nephron development, and Metabolism of Angiotensinogen to Angiotensins. As an inflammatory cytokine with multiple biological effects, TNF exerts its biological activity by binding and activating two distinct receptors, TNFR1 and TNFR2 (56). Previous research has confirmed that the binding of TNF to TNFR1 can promote inflammatory responses. This study suggests that TNF may also activate the atypical NF-κB pathway through binding to TNFR2, further enhancing the expression of inflammatory genes and the production of various inflammatory cytokines and chemokines, thus initiating and amplifying inflammatory responses (57). Additionally, TNF receptors and casein kinase II (CK2) contribute to atrial fibrosis, ion channel dysfunction, and electrical conduction abnormalities by activating inflammatory signaling pathways (such as NF-κB) and profibrotic cascades. Both also induce oxidative stress, which activates the NLRP3 inflammasome and promotes the release of proinflammatory cytokines such as IL-1β, thereby exacerbating myocardial injury and fibrosis. Together, these mechanisms drive atrial structural remodeling and electrical instability, facilitating the initiation and progression of atrial fibrillation (55).We have also found a correlation between the occurrence of atrial fibrillation and the WNT signaling pathway. Previous studies have confirmed the association of this pathway with myocardial fibrosis (58–60). Dishevelled protein (DVL) plays a critical regulatory role in both the canonical Wnt/β-catenin signaling pathway and the non-canonical Wnt/planar cell polarity (PCP) signaling pathway. By promoting the differentiation of myofibroblasts, this leads to myocardial fibrosis and subsequently the occurrence of atrial fibrillation (61). The RAS system is also one of the factors by which hyperuricemia promotes the occurrence of atrial fibrillation. Hyperuricemia can directly or indirectly activate the RAS system’s ACE/AII/AT1 axis (62), stimulating oxidative stress and cytokine release, promoting inflammatory responses, and also participating in cardiac electrical remodeling, inducing morphological changes in atrial cardiomyocytes. Additionally, it may stimulate epicardial fat accumulation and inflammation, thereby directly or indirectly affecting the occurrence of atrial fibrillation (63). Moreover, clinical evidence has shown that classical RAS inhibitors (ACE-Is and ARBs) can reduce the incidence Additionally, MECP2 regulation of transcription of neuronal ligands, condensation of prometaphase chromosomes, and receptor-mediated mitophagy may play a role in the onset of atrial fibrillation induced by hyperuricemia, but the specific mechanisms remain unknown and require further investigation. Through protein-level analysis, we have unveiled the potential mechanisms by which HUA induces AF, identifying a link between purine metabolic disorder and the onset of AF, potentially offering new targets for the prevention and treatment of atrial fibrillation with significant clinical implications.

In recent years, accumulating studies have elucidated the association between hyperuricemia and AF. Mechanistically, activation of the NLRP3 inflammasome has emerged as a key contributor to AF pathogenesis. Hyperuricemia promotes the release of proinflammatory cytokines such as IL-1β by activating the NLRP3 inflammasome, leading to atrial fibrosis and structural remodeling (55, 64). Additionally, xanthine oxidase (XO)–mediated oxidative stress contributes to autophagy dysregulation and impaired calcium handling, further enhancing AF susceptibility (65). From a clinical intervention perspective, urate-lowering therapies—such as allopurinol and febuxostat—have become a focus of AF-related research. Both drugs are XO inhibitors but act via distinct mechanisms. While allopurinol has shown potential in reducing AF risk among older adults, febuxostat has been associated with an increased risk of AF in the same population, suggesting the need for individualized treatment decisions (66). Moreover, the impact of hyperuricemia on AF appears to vary significantly by ethnicity, potentially linked to polymorphisms in the URAT1 gene. A stronger association observed in female patients may be attributable to estrogen-mediated regulation of XO activity (67, 68). These findings underscore the importance of conducting mechanistic studies in more diverse, multi-ethnic cohorts to better understand the relationship between uric acid and AF. Targeting key nodes within the NLRP3 inflammasome or purine metabolism pathways may provide a theoretical foundation for developing more selective therapeutic strategies.

From a translational medicine standpoint, the 17 mediator proteins identified in this study offer promising utility as blood-based biomarkers for early diagnosis and risk stratification. Moreover, these key proteins may serve as targets for novel therapeutics or the repurposing of existing drugs. Stratified intervention strategies could also be developed based on protein expression profiles. Future research should focus on validating the clinical utility of these biomarkers through multicenter cohort studies and elucidating the causal mechanisms of candidate proteins using organoid models or gene-editing technologies, thereby providing an experimental basis for rational target selection.

This study is innovative in the following ways. To our knowledge, this is the first study to apply mediation analysis to explore the causal relationship between serum uric acid levels and the onset of atrial fibrillation, including the mediation pathways involved. Furthermore, this study was conducted using large-sample pooled data from genome-wide association studies. Confounding-related SNPs were eliminated from the statistical model, and multiple sensitivity analysis techniques were employed to ensure the robustness and reliability of the causal conclusions. Our conclusions are subject to the following limitations. Initially, our study population focused on Europeans; hence, the conclusions are presently not generalizable to a broader demographic. Furthermore, the use of aggregate data rather than individual-level data precludes stratification by variables such as gender. This limitation is particularly important given that multiple studies have demonstrated a positive association between HUA and AF risk in both men and women, with significant sex-specific differences (68, 69), underscoring the need for future sex-stratified investigations. The assumption of a linear causal relationship inherent in the ratio estimation method means that a non-linear relationship between hyperuricemia and the incidence of AF cannot be dismissed. Lastly, individual channeling is an unavoidable aspect of Mendelian Randomization (MR) studies. However, it is important to note that the conclusions of the MR study remain valid as long as the SNPs used in this research satisfy the three assumptions of an instrumental variable.

Overall, our innovative findings indicate that impairments in β-cells due to abnormal states and activities of transcription factor HNF4α are a significant cause of atrial fibrillation induced by hyperuricemia, indirectly corroborating the frequent co-occurrence of atrial fibrillation in diabetic patients.

## 5 Conclusion

Hyperuricemia is closely associated with the incidence of atrial fibrillation. Seventeen plasma proteins, including Hepatocyte nuclear factor 4-alpha with mediating effects, play a critical role in the process by which hyperuricemia promotes atrial fibrillation, elucidating the potential link between purine metabolic disorder and AF pathogenesis, and offering potential targets for future prevention and treatment of atrial fibrillation.

## Data Availability

The raw data supporting the conclusions of this article will be made available by the authors, without undue reservation.
