# Supplementary material for: Decoding the mystery between hyperuricemia and atrial fibrillation: new causal links through mediating proteomics
Source: Front Endocrinol (Lausanne). 2025 May 21;16:1429465. doi: 10.3389/fendo.2025.1429465 (PMC12133553; doi:10.3389/fendo.2025.1429465)
Supplement: Supplementary file 1 [file DataSheet1.pdf]

Supplementary File 1. Extended Description of the Cohorts Used in the Serum Uric Acid GWAS

Study Name	Study Design	Total Genotyped Sample Size	Exclusion Criteria for Study Participation or Disease Enrichment	Exclusions	Population Stratification	UA Measurement and QC	Gout Definition	Key Study References
Discovery Studies								
AGES Reykjavik Study	Prospective, population-based	3,219 of European ancestry	none	none	All individuals from Iceland, with no significant stratification within the population.	Serum urate was measured at the Icelandic Heart Association using the Roche-Hitachi P-Module instrument with Roche uricase method. The coefficient of variation for the urate assay was 4.3%.	Gout was determined from a positive answer on a questionnaire or if the participant was on allopurinol treatment at a study visit.	Harris et al. (2007)[2]
Amish Studies	Founder "healthy" population based[3].	European ancestry	none	none	NA	Serum UA levels drawn at the screening exam were assayed by Quest Diagnostics (Baltimore, MD) and measured to the nearest 0.1 mg/dl[4].	NA	Mitchell et al. (2008)[3], McArdle et al. (2008)[4]
Atherosclerosis Risk in Communities (ARIC) Study	Prospective, population-based[5]	9,713 of European ancestry	none	Of the 9713 genotyped individuals of European ancestry, we excluded 658 individuals based on discrepancies with previous genotypes, disagreement between reported and genotypic sex, one randomly selected member of a pair of first-degree relatives, or outlier based on measures of average DST or more than 8 SD away on any of the first 10 principal components.	Two principal components were associated with uric acid measurements and included as covariates in the regression.	UA was measured using the uricase method[6] at study visit 1. Repeated measurements of UA in 40 individuals, taken at least one week apart, yielded a reliability coefficient of 0.91, and the coefficient of variation was 7.2%[7].	Gout was defined by self-report at study visit 4 based on the question "did a doctor ever tell you that you had gout?".	ARIC (1989)[5], Iribarren et al. (1996)[6], Eckfeldt et al. (1994)[7]
Austrian Stroke Prevention	Prospective, population-based	923 genotyped Caucasians	no history or signs of stroke and dementia	Of the 923 genotyped individuals we excluded 67 subjects based on excess	Age and sex were included as covariates in the	UA was measured using the uricase method on a Hitachi	A subject was defined as having gout at	Schmidt et al. (1994)[8]

Nature Genetics doi:10/1038/ng.2500

The cohort details described above are derived from Supplementary Table 2 of the original publication. For comprehensive information, refer to:
Köttgen A, Albrecht E, Teumer A, et al. Genome-wide association analyses identify 18 new loci associated with serum urate concentrations. Nat Genet. 2013;45(2):145–154. doi:10.1038/ng.2500

Study (ASPS)		living in the city of Graz, Austria		autosomal heterozygosity, mismatch between called and phenotypic gender, or by being outliers identified by the IBD analysis. The final population for genetic analysis comprised 856 subjects. Additionally serum urate was not available in 3 cases.	regression	917 chemical analyzer at study visit 1. Reproducibility was assessed in 21 subjects and revealed a variation coefficient of 1.7%	study visit 1 if he/she reported a history of elevated uric acid levels and was currently treated for hyperuricemia.	
Australian Twin-Family Study (AUSTWIN)	Population-based, twin-pairs and their families	11,520 of European ancestry	none	Samples were excluded for less than 95% of SNPs successfully typed, sex or Mendelian errors, Non-European ancestry	Two principal components were included as covariates in the regression.	Serum uric acid was measured with the uricase method on a Roche 917 or Modular P analyser.	NA	Whitfield et al. (2002)[9], Middelberg et al. (2007)[10], Benyamin et al. (2009)[11]
Baltimore Longitudinal Study of Aging (BLSA)	Prospective, population-based	1,230	none	Of the 1230 genotyped subjects, genetic relatedness was assessed using PCA analysis using the HapMap population as reference. Out of the 857 subjects of European ancestry, 5 subjects were excluded for low genotyping (< 98.5%), 4 subjects were removed for sex misspecification. From the 848 subjects with European ancestry passing quality control, 718 subjects with uric acid data was used for this study.	Use top two principle components included as covariates in the regression model.	UA was measured using the uricase method (Johnson and Johnson, VITROS chemistry system).	NA	Shock et al. (1984)[12]
The BRItish Genetics of HyperTension (BRIGHT) study	Hypertensive cases from the BRIGHT study resource4.	1,743	Control exclusion criteria included BMI>35, diabetes, secondary hypertension or a co-existing illness. Blood pressure was measured using the OMRON-705CP blood pressure monitor.	Of 2000 cases typed, we excluded 257 people with poor genotype quality.	NA	Non-fasting blood samples were obtained from study participants and UA analyses was carried out on frozen serum stored at − 20 C. UA concentrations were measured using an uricase method on a Hitachi auto-analyser).	NA	Caulfield et al. (2003)[13]

Coronary Artery Disease Risk in Young Adults (CARDIA)	Prospective, population-based	1,725 of European Ancestry	none	1 sex mismatch; 3 outliers in PCA; 1 discordant genotype	4 principal components included as covariates; none associated with UA	Serum uric acid was measured by the uricase method at multiple visits. The coefficient of variation of uric acid was 2.6%; the split sample technical error was 4.6%.	NA	Friedman et al. (1988)[14], The data collection forms used at each exam as well as the CARDIA protocols are available from the CARDIA website: http://www.cardia.dopm.uab.edu/em_dacf.htm
The Cardiovascular Health Study (CHS)	Prospective, population-based	3,329 CHS Caucasian participants	1908 persons were excluded due coronary heart disease, congestive heart failure, peripheral vascular disease, valvular heart disease, stroke or transient ischemic attack.	The present report is based upon genotyping results from 3,329 CHS Caucasian participants, who were free of clinical cardiovascular disease at baseline, consented to genetic testing, and had DNA available for genotyping. Genotypes were called using the Illumina BeadStudio software. Genotyping was successful in 3,291 persons.	Study sites (clinic sites) were included as covariates in the regression to account for population stratification.	Serum uric acid concentrations were measured at the baseline visit using the Kodak Ektachem 700 Analyzer with reagents (Eastman Kodak, Rochester, NY). The final study sample with available genotype and phenotype data consisted of 3,252 individuals for the analyses of uric acid.	Intake of previous or current gout-specific medication: colchicine, probenecid, or allopurinol. Total of 3,192 individuals for the analyses of gout.	Fried et al. (1991)[15]
Cohorte Lausannoise (CoLaus) Study	Population based	5,636 of European ancestry	none	Individuals with call rate below 90% were excluded. The younger of 1st/2nd degree related pairs were removed from the analysis.	First two ancestry principal components were used as covariates.	Serum uric acid was measured by uricase-PAP (1.0% - 0.5% maximum inter and intra-batch coefficients of variation).	Gout was indirectly defined: People taking allopurinol or colchicine.	Firmann et al. (2008)[16]
CROATIA-KORCULA	Cross-sectional, population-based	971	none	898 individuals left after QC based on genotyping quality, sex and ancestry check	None of the first 3 principal components strongly associated with uric acid; relatedness of participants taken into account using a mixed linear model with the polygenic effect set as random effect.	UA was measured using the uricase UV photometry method in "Labor Centar" biochemical lab, Bukovcev trg 3, 10000 Zagreb Croatia (www.laborcentar.hr).	Gout case based on self-report and medication; 46 cases	Zemunik et al. (2009)[17]
CROATIA-SPLIT	Cross-sectional, population-based	535	none	499 individuals left after QC based on genotyping quality, sex and ancestry check.	None of the first 3 principal components strongly associated	UA was measured using the uricase UV photometry method in "Labor Centar"	Gout case based on self-report and medication;17	Rudan et al. (2009)[18]

Nature Genetics doi:10/1038/ng.2500

					with uric acid; relatedness of participants taken into account using a mixed linear model with the polygenic effect set as random effect.	biochemical lab, Bukovcev trg 3, 10000 Zagreb Croatia (www.laborcentar.hr).	cases	
CROATIA-VIS	Cross-sectional, population-based	991	none	924 individuals left after QC based on genotyping quality, sex and ancestry check	None of the first 3 principal components strongly associated with uric acid; relatedness of participants taken into account using a mixed linear model with the polygenic effect set as random effect.	UA was measured using the uricase UV photometry method in "Labor Centar" biochemical lab, Bukovcev trg 3, 10000 Zagreb Croatia (www.laborcentar.hr). A subset of 774 samples had also been measured independently in the Institute for Clinical Chemistry and Laboratory Medicine, University Hospital Regensburg, Germany. Pearson correlation between the two urate measurements was 94% .	Gout case based on self-report and medication; 58 cases used in analysis	Vitart et al. (2006)[19]
Data from the Epidemiologica l Study on the Insulin Resistance Data from the Epidemiologica l Study on the Insulin Resistance Syndrome (DESIR) Study	Controls for the study of T2D and obesity selected from a population-based study.	716 of European ancestry	none	Using the STRUCTURE software, we identified 4 individuals of non-European ancestry. In order to minimize admixture bias in the rest of the DESIR participants, we excluded these individuals before analyses	none	UA was measured using the uricase method [20] at study visit 1. Repeated measurements of UA in 40 individuals, taken at least one week apart, yielded a reliability coefficient of 0.91, and the coefficient of variation was 7.2% [21].	NA	Balkau et al. (1997)[20], Vernay et al. (2004)[21]
European Prospective Investigation of Cancer (EPIC) Norfolk Study	Prospective, population-based, case-cohort design consisting of a random sample	3,850 of European ancestry	none	We excluded individuals who were duplicated samples DNA concordance >99%, cryptically related, related individuals DNA concordance >70% and	The 3552 individuals who were used for GWAS repeatedly showed no evidence of	Out of these individuals 2856 had uric acid measured, marked as serum L:89 H:1785 umol/L Olympus AU640.	Gout was defined as gout mentioned on hospital discharge records (ICD10	Day et al. (1999)[22], *http://www.srl.cam.ac.uk/epic/about/

	(cohort) of 2566 participants at baseline and 1284 obese cases [22],*			<99%, ethnic outliers, and heterozygosity <23% or >30%. In the discovery analysis only controls were used. Obese cases were used for replication.	population stratification. Consequently, we have not adjusted for population stratification.		M10, between 1997-2008) or self-reported gout specific medication (colchicine, probenecid or allopurinol) at any follow-up.	
Erasmus Ruchphen Family (ERF) Study	Family based	2,385	none	none	Score test for association in related people implemented in R package GenABEL was used to control for family related ness.	UA concentrations were measured using an uricase/peroxidase method (DVIA1650-Autoanalyzer, Siemens Healthcare Diagnostics)	Gout was defined by intake of gout-specific medication: colchicine, probenecid or allopurinol	Pardo et al. (2005)[23]
Estonian Genome Center of University of Tartu (EGCUT)	Prospective, population-based	931 of European ancestry	none	Low genotyping quality (call rate <98%, MAF <1%, HWE p-value 10E-6); disagreement between reported and genotypic sex, one randomly selected member of a pair of first-degree relatives	Three principal components were associated with uric acid measurements and included as covariates in the regression.	UA was measured using the uricase method	Diagnosed at least by family doctor in ICD10 coding M.10	Nelis et al. (2009)[24], Metspalu et al. (2004)[25]
Family Heart Study (FamHS)	Population family-based [26]	4,135 of European ancestry		Quality control was performed before imputation. To assess Mendelian errors, we ran LOKI on our family data and removed 5,035 SNPs with Mendelian errors. We also removed 2 individuals that had an unaccepted number of Mendelian errors. As a final familial QC check, we used GRR software to check familial relationships based on IBS. Quality control procedures for SNPs included cleaning SNPs reported by Illumina as uninformative and unavailable on successive arrays (n=13,844), removing SNPs due to deviations from Hardy-Weinberg equilibrium	Ten principal components (EIGENSTRAT) were estimated using the genotype data of the largest sample of independent subjects (N= 753) and then applied to the family members. These principal components were included in the adjustment procedure of uric acid using stepwise regression analysis and held if they were significant at 5% level.	Uric acid was measured by a thin film adaptation of an uricase enzymatic method using the Vitros analyzer (Johnson & Johnson Clinical Diagnostics, Inc. Rochester NY 14650).	NA	Higgins et al. (1996)[26], Neogi et al.. (2011)[27], Neogi et al. (2009)[28], Tang et al. (2006)[29], Tang et al. (2003)[30], Wilk et al (2000)[31]

Nature Genetics doi:10/1038/ng.2500

				(p<1E-06) or SNPs with minor allele frequency <1% or >99% (n=22,088), and removing SNPs that are available in our data but not in HapMap (n=1,509). Additionally, 21 SNPs were designated as ambiguous and removed. After these quality control procedures, genotypes are available for 4,135 European American (EA) subjects with imputed genotypes for ~2.5 million SNPs.				
The Framingham Heart Study	Prospective, family based	9,274	none	Individuals with a sample call rate <97%, or heterozygosity > ±5 SD from the mean are excluded from association analyses.	Principal components of the genotypes of 550K SNPs were computed using the Eigenstrat software 5, and none of the first 10 components were found association with either urate levels or gout using a Bonferroni correction on alpha of 0.05, which indicated that there is little population admixture for these two traits and therefore no need to adjust for admixture in GWAS.	Serum urate was measured at the first examination cycle of each cohort using an autoanalyzer with a phosphotungstic acid reagent	Gout was ascertained via self-report in the Offspring subjects during exam cycles 3-7, and the first exam of the Third Generation	Dawber et al. (1963)[32], Crowley et al. (1964)[33]
Health 2000	Population-based	2,123 Finns	none	Samples with discrepancy between reported and genotypic sex were excluded. For pairs with pi_hat > 0.2 one of the pairs was excluded. Individuals with 0.05 < pi_hat < 0.2 to many other individuals were excluded.	NA	Uricase method, a colorimetric enzymatic method (Thermo Fisher Scientific, Vantaa, Helsinki).	NA	http://www.terveys2000.fi/doc/methodologyrep.pdf

Nature Genetics doi:10/1038/ng.2500

InCHIANTI study	Prospective, population-based	1,230 European ancestry	none	Of the 1231 genotyped subjects, 22 subjects were removed based on genotyping completeness (<97%), low heterozygosity (<0.3), or sex misspecification. 1205 subjects with uric acid data was used for the analysis.	Genomic Control	Plasma UA (mg/dl) was measured using an enzymatic-colorimetric method (Roche Diagnostics, GmbH, Germany). The lower limits of detection were 0.2 mg/dl, range 0.2–25.0 mg/dl, intra- assay and inter assay coefficients of variation (CV) were 0.5 and 1.7%, respectively.	NA	Ferrucci et al. (2000)[34]
INCIPE	Randomly chosen from the lists of patients of 62 randomly selected general practitioners (GPs) based in four geographical areas in the Veneto region, Northern Italy.	942 from Northern Italy	none	992 genotyped individuals (then 50 removed). Disagreement between reported and genotypic sex, one randomly selected member of a pair of first-degree relatives	From same geographical area	UA was measured using the UV uricase method; the between series CV is 1.5%	NA	Gambaro et al. (2010)[35]
INGI-Carlantino	Population-Based	659	none	Removed people with call rate <0.95 or too high IBS or heterozigosity. Removed people that did not pass sex chromosome checks or were < 18 years of age.	Corrected using mixed model regression analysis.	UA was measured with the colorimetric method using Targa 3000 from Biotecnica Instruments.	NA	Tepper et al. (2008)[36]
INGI-CILENTO	Population-Based study with pedigree information	859	none	Of the 859 participants who underwent genotyping, none was excluded	none	UA was measured using an enzymatic method.	NA	Ciullo et al. (2006)[37], Colonna et al. (2007)[38], Ciullo et al. (2008)[39], Sala et al. (2008)[40], Traglia et al. (2009)[41], Heid et al. (2009)[42], Colonna et al. (2009)[43], Bedin et al. (2009)[44], Siervo et al. (2010)[45]
INGI-FVG	Population-Based	1,471	none	Removed people with call rate <0.95 or too high IBS or heterozigosity. Removed people that did not pass	Corrected using mixed model regression analysis.	UA was measured with the colorimetric method using Targa 3000 from Biotecnica	NA	Girotto et al (2011)[46]

Nature Genetics doi:10/1038/ng.2500

				sex chromosome checks or were < 18 years of age.		Instruments		
INGI-Val Borbera	Family Population-based	1,665	none	Of the 1665 participants who underwent genotyping, we made the following exclusions: sample call rate <95% (n=1)	NA	UA was measured using HITACHI 917 ROCHE and Unicel Dx-C 800 BECKMAN	Gout was defined by self-report at study visit, or intake of gout specific medication	Traglia et al. (2009)[41]
KORA F3	Population-based	1,644	none	Only subjects with overall genotyping efficiencies of at least 93% were included. In addition the called gender had to agree with the gender in the KORA study database.	none	Non-fasting blood samples were obtained from study participants. UA analyses were carried out on fresh samples. UA concentrations were measured using an uricase method (URCA Flex, Dade Behring).	Current intake of urate-lowering medication	Wichmann et al. (2005)[47]
KORA F4	Population-based	1,814	none	Only subjects with overall genotyping efficiencies of at least 93% were included. In addition the called gender had to agree with the gender in the KORA study database.	none	Fasting blood samples were obtained from study participants. UA analyses were carried out on fresh samples. UA concentrations were measured using an uricase method (URCA Flex, Dade Behring).	Current intake of urate-lowering medication	Wichmann et al. (2005)[47]
LBC1936	Retrospective and prospective community-based cohort study[48]	1,005 of European ancestry	none	Individuals with a disagreement between genetic and reported gender were removed (n=12). Relatedness between subjects was investigated and, for any related pair of individuals, one was removed (PI_HAT (proportion of IBD) > 0.25, n=8). Samples with a call rate ≤ 0.95 (n=16), and those showing evidence of non-European descent by multidimensional scaling, were also removed (n=1).	None of the four extracted principal components were associated with uric acid measurements so were not included in the model.	Serum uric acid was determined using the VITROS URIC DT slide method performed using the VITROS URIC DT slide and the VITROS Chemistry products DT Calibrator Kit on VITROS DT60/DT60 II Chemistry systems (VITROS). This was performed at the Combined Biochemistry and Haematology Labs, Western General Hospital, Edinburgh.	Gout was defined by self-report based on the question “Any other disease or health problem?” or evidence of allopurinol in current medication.	Deary et al. (2007)[48], Houlihan et al. (2010)[49]

Nature Genetics doi:10/1038/ng.2500

LifeLines Cohort Study	Prospective, population-based	3,367 of European ancestry	none	Of the 3900 genotyped individuals, we excluded 533 individuals based on discrepancies with previous genotypes, disagreement between reported and genotypic sex, one randomly selected member of a pair of first-degree relatives, and non-European ancestry	NA	Uric acid was measured on a Roche/Hitachi Modular System (Roche Diagnostics GmbH), by the uricase/peroxida enzymatic method	NA	Stolk et al. (2008)[50]
London Life Sciences Population (LOLIPOP) study, LOLIPOP_EW610	Prospective, population-based	945		Duplicates, gender discrepancy, contaminated samples, relatedness	The first ten principal components were used as covariates in the regression.	Venous blood was collected into 5.0ml BD Vacutainer SST II Advance tube. Serum urate measurements were measured using the uricase method on Roche/Hitachi Cobas C 501 systems (USA).	NA	
London Life Sciences Population (LOLIPOP) study, LOLIPOP_EW_A	Prospective, population-based	878		Duplicates, contaminated samples, relatedness, samples already in EW610	The first ten principal components were used as covariates in the regression.	Venous blood was collected into 5.0ml BD Vacutainer SST II Advance tube. Serum urate measurements were measured using the uricase method on Roche/Hitachi Cobas C 501 systems (USA).	NA	Yuan et al. (2008)[51]
London Life Sciences Population (LOLIPOP) study, LOLIPOP_EW_P	Prospective, population-based	1,006		Duplicates, contaminated samples, samples already in EW610 and EW_A	The first ten principal components were used as covariates in the regression.	Venous blood was collected into 5.0ml BD Vacutainer SST II Advance tube. Serum urate measurements were measured using the uricase method on Roche/Hitachi Cobas C 501 systems (USA).	NA	Kooner et al. (2008)[52]
Ludwigshafen Risk and Cardiovascular Health Study (LURIC)	Prospective, case-control (CAD)	963	any acute illness other than ACSs, any chronic disease where non-cardiac disease predominated a history of malignancy within the past	Individuals with genotyping call rates below 0.96 were removed.	none	UA was measured using a photometric colour test (Harnsäure Farb-Reagenz, Greiner, Germany) on a Hitachi 717 at study entry.	Gout was defined by the recorded intake of anti-gout medication	Winkelmann et al. (2001)[53]

Nature Genetics doi:10/1038/ng.2500

			five years					
MICROS	Cross-sectional, population-based	1,345	none	1,268 individuals left after QC based on genotyping quality, sex and ancestry check.	None of the first 3 principal components strongly associated with uric acid but village of origin kept as cofactor; relatedness of participants taken into account using a mixed linear model with the polygenic effect set as random effect.	UA was measured using the uricase /peroxidase method.	Self-reported; 39 cases	Pattaro et al. (2007)[54]
Netherlands Study of Depression and Anxiety (NESDA)	Longitudinal cohort study of individuals with depressive and/or anxiety disorder	1,862 of western-European ancestry	Individuals were almost all cases with major depression or anxiety disorder (n=1705)	Ethnic outliers, XO and XXY samples, and samples with a call rate <95%, high genome-wide homo- or heterozygosity, excess IBS were excluded	none	UA was measured by enzymatic colorimetric test (uricase method, Roche Modular system). The coefficients of variation, over the complete measurement period, were 1.6% at a level of 0.25 mmol/l and 1.2% at a level of 0.55 mmol/l.	NA	Penninx et al. (2008)[55], Sullivan et al. (2009)[56]
NSPHS	Cross-sectional, population-based	700	none	656 individuals left after QC based on genotyping quality, sex and ancestry check	None of the first 3 principal components strongly associated with uric acid; relatedness of participants taken into account using a mixed linear model with the polygenic effect set as random effect.	UA was measured using the uricase /peroxidase method.	NA	Igl et al. (2010)[57]
ORCADES	Cross-sectional, population-based	920	of non-orcadian ancestry	889 individuals left after QC based on genotyping quality, sex and ancestry check.	None of the first 3 principal components strongly associated with uric acid; relatedness of participants taken	UA was measured using the uricase /peroxidase method in the Balfour Hospital, Kirkwall, UK. A subset of 718 samples had also been measured	Gout case based on self-report and medication; less than 50 cases	McQuillan et al. (2008)[58]

Nature Genetics doi:10/1038/ng.2500

					into account using a mixed linear model with the polygenic effect set as random effect.	independently in the Institute for Clinical Chemistry and Laboratory Medicine, University Hospital Regensburg, Germany. Pearson correlation between the two urate measurements was 99%.		
PREVEND	Prospective, population-based [59]	4,016 of European ancestry	none	Of the 4,016 genotyped individuals, we excluded 148 individuals based on discrepancies with previous genotypes, disagreement between reported and genotypic sex, first-degree relatives, or outlier based PCA.	none	Uric acid was measured in plasma and urine with the uricase PAP method as described previously (MEGA, Merck, Darmstadt, Germany). [60]	NA	Hillege et al. (2002)[59]
Procardis	Case-Control study of CAD	3,742	none	Dataset was prefiltered for individuals with success rate <95%, ancestory outliers on PCA, heterozygosity, IBC	Country of Origin was added as a covariate, population stratification was checked using PCA but was not adjusted for beyond Country of Origin.	Measured using uricase method in hospital clinical lab	NA	Broadbent et al. (2008)[61]
RS-I	Prospective, population based	5,974	none		none	Serum urate was measured at the baseline visit using a Kone Diagnostica reagent kit and autoanalyzer.	Using a computer network of pharmacies, data on medication prescription use was abstracted from pharmacies in the study region that registers all medication prescriptions beginning January 1, 1991. Participants	Hofman et al. (1991)[62], Hofman et al. (2009)[63]

Nature Genetics doi:10/1038/ng.2500

							receiving medication (allopurinol, benzbromarone, colchicine, and probenecid) were considered gout cases.	
RS-II	Prospective, population based	2,157	none		none	Serum urate was measured at the baseline visit using a Kone Diagnostica reagent kit and autoanalyzer.	Using a computer network of pharmacies, data on medication prescription use was abstracted from pharmacies in the study region that registers all medication prescriptions beginning January 1, 1991. Participants receiving medication (allopurinol, benzbromarone, colchicine, and probenecid) were considered gout cases.	Hofman et al. (1991)[62], Hofman et al. (2009)[63]
SardiNIA Study	Population-based study in Sardinia. The SardiNIA study consists of 6,148 individuals, males and females, ages 14-102 y, that were recruited from a cluster of four towns in the Lanusei	4,694	none	none	none	During physical examination, a blood sample was collected in the morning after the participants had been fasting for at least 12 h and after sitting for 15 min and divided into two aliquots. One was used for genomic DNA extraction and the second aliquot to characterize several	Diagnosis of gout was self-report by the participants during anamnesis based on the question "Have you ever been diagnosed with gout?"	Pilia et al. (2006)[64], Li et al. (2007)[65], Sanna et al. (2008)[66]

Nature Genetics doi:10/1038/ng.2500

	Valley of Sardinia. Samples have been characterized for several quantitative traits and medical conditions, including serum urate.					blood phenotypes, including evaluation of serum UA. UA (mg/dl) was measured using enzymatic–colorimetric methods (Bayer) The lower limits of detection were 0.2 mg/dl, range 0.2–25.0 mg/dl, intra-assay and inter assay coefficients of variation were equal to 0.5% and 1.7%, respectively.		
Study of Health in Pomerania (SHIP)	Population-based	4,081 of European ancestry	none	24 individuals identified as duplicated or with reported/genotyped gender mismatch	none	Uricase method, a colorimetric enzymatic method (Uric acid PAP, Boehringer) from non-fasting, fresh serum	Gout was defined by self-report at study visit on the question: Did you have any of the following diseases in the last 12 months? Gout or increased uric acid levels?	John et al. (2001)[67], Völzke et al. (2011)[68]
SOCCS	Colorectal cancer case control study, population-based	2,024	none	1,984 individuals after QC, 1,105 of whom had uric acid phenotypes.	No PCs of ancestry included in analysis	UA was measured using the uricase /peroxidase method.	Self-reported information "Up until a year ago had you ever had any other serious illness, chronic condition or mental health condition?". 3 cases. Less than 50 cases.	Tenesa et al. (2008)[69]
Sorbs	Population-based	1,020	46 individuals excluded as they were on a medication that lowers serum uric acid	ethnic outliers, duplicates, and gender mismatches	Estimation of kinship matrix to take account of relatedness.	enzymatic color test (Roche Diagnostics, Inc)	NA	Tönjes et al. (2009)[70], Tönjes et al. (2010)[71], Veeramah et al. (2011)[72]
TwinsUK	Twins	5,654 of European ancestry	none	Samples: Exclusion criteria were: (i) sample call rate <98%, (ii) heterozygosity	Estimation of kinship matrix to take account of	Ektachem/Vitros system, Johnson & Johnson Clinical	NA	Moayyeri et al. (2012)[73]

Nature Genetics doi:10/1038/ng.2500

				across all SNPs >2 s.d. from the sample mean; (iii) evidence of non-European ancestry as assessed by PCA comparison with HapMap3 populations; (iv) observed pairwise IBD probabilities suggestive of sample identity errors; (v). We corrected misclassified monozygotic and dizygotic twins based on IBD probabilities.	relatedness.	Diagnostics		
Women's Genome Health Study	Prospective, population based	23,294 with verified European ancestry	none	none	No principal component was significant.	NA	Gout was defined by self-report on any follow-up questionnaire based on the question "In the past year, have you been diagnosed with gout?" ICD9 codes 274.0, 274.1, 274.8, 274.9	Ridker et al. (2007)[74]
Young Finns Study	Birth cohort follow-up	2,443 Finns	none	Samples with discrepancy between reported and genotypic sex were excluded. For pairs with pi_hat > 0.2 one of the pairs was excluded. Individuals with 0.05 < pi_hat < 0.2 to many other individuals were excluded.	none	Uricase method, a colorimetric enzymatic method (Thermo Fisher Scientific, Vantaa, Helsinki).	NA	Raitakari et al. (2008)[75]
In Silico Replication Studies								
EPIC - cases	See description above.							
GSK cases/controls	Case-control study for unipolar depressive disorder	819 cases/ 851 controls of European ancestry	GSK cases: patients with unipolar recurrent depression, exclusion criteria: presence of	MDS-analysis revealed no outliers (more than 8SD away on any of the first 10 principal components): after QC 819 cases/851 controls.	No principal component was associated with uric acid so none was included as covariates. No principal component was	UA was measured using the uricase method (Roche/Hitachi cobas c system, UA ver.2).	Information on gout was not obtained.	Lucae et al. (2006)[76], Kloiber et al. (2010)[77], Kohli et al. (2011)[78]

			manic or hypomanic episodes, mood incongruent psychotic symptoms, lifetime diagnosis of drug abuse and depressive symptoms secondary to alcohol or substance abuse or dependence or to a medical illness or medication GSK controls: exclusion criteria: anxiety and affective disorders.		associated with uric acid so none was included as covariate.			
Gutenberg Health Study (GHS I + II)	Population-based	4860 (3422 (GHS I) + 1438 (GHS II))	age below 35 and above 74	Of the 4860 we excluded 685 (426 + 259) based on a call rate less than 97 %, a rate of heterorzygosity 3 standard deviations away from the mean, disagreement between reported and genotypic sex, estimated IBD > 0.25, IBS based principal components.	none	UA was measured using the uricase method at study visit during routine meausrements. Intra coefficient of variation (CV) was 0% at a mean value of 4.9 mg/dL and 0.44% at a mean value of 9.52 mg/dL, the inter CV% was 2.25% at a mean value of 4.9 mg/dL, and 0.97% at a mean value of 9.4 mg/dL.	NA	Zeller et al. (2010)[79], Wild et al. (2010)[80], Wild et al. (2011)[81]
Hunter Community Study (HCS)	Prospective, population-based	1,230 of European ancestry	none	Individuals were excluded for genotype call rate <95%, discrepancies between clinical and inferred gender, one randomly selected member of a pair of first- or second-degree relatives or clear evidence of non-European	No principal components were associated with uric acid, and were not included as covariates.	The HAPS pathology service did the urate measurements. They are a NATA accredited lab and meet national standards for quality assurance.	Gout was defined by self-report use of "Gout medication".	McEvoy et al. (2010)[82]

				ancestry in Eigenstrat PCA.				
LifeLines Cohort Study	Prospective, population-based	5,031 of European ancestry	none	none	NA	Uric acid was measured on a Roche/Hitachi Modular System (Roche Diagnostics GmbH), by the uricase/peroxidase enzymatic method.	NA	Stolk et al. (2008)[50]
Ludwigshafen Risk and Cardiovascular Health Study (LURIC)	Prospective, case-control (CAD)	1,960	any acute illness other than ACSs, any chronic disease where non-cardiac disease predominated a history of malignancy within the past five years	Individuals which were part of the discovery analysis were removed. Samples were also removed because of gender discrepancy, relatedness or low call rate (<90%).	Sample HD (Heidelberg) n=1156 and GZ (Graz) n=804 were analyzed separately.	UA was measured using a photometric colour test (Harnsäure Farb-Reagenz, Greiner, Germany) on a Hitachi 717 at study entry.	Gout was defined by the recorded intake of anti-gout medication	Winkelmann et al. (2001)[53]
MARS cases	Case-control study for depressive disorder	643 cases of European ancestry	MARS cases: patients with depressive episode, exclusion criteria: depressive disorders caused by a medical or neurologic condition and alcohol or substance dependence.	MDS-analysis revealed 7 outliers (more than 8SD away on any of the first 10 principal components): after QC: 636 cases.	No principal component was associated with uric acid so none was included as covariates. No principal component was associated with uric acid so none was included as covariate.	UA was measured using the uricase method (Roche/Hitachi cobas c system, UA ver.2).	Information on gout was not obtained.	Kohli et al. (2011)[78]
Ogliastra Genetic Park - Talana	Population-based study with pedigree information	860	none	none	none	Uric acid levels were measured using the uricase method with an automated TARGA BT-3000 Chemistry Analyser	Gout was defined by self-report at study visit based on the question "did a doctor ever tell you that you had gout?".	Portas et al. (2010)[83], Biino et al. (2010)[84], Tore et al. (2011)[85]
Study of Health in Pomerania -	Population-based	986 of European ancestry	none	array call rate < 94%, individuals identified as duplicated or with	none	UA was measured from non-fasting, fresh serum. An	Gout was defined by self-report at study	John et al. (2001)[67], Völzke et al. (2011)[68]

Nature Genetics doi:10/1038/ng.2500

Trend (SHIP-Trend)				reported/genotyped gender mismatch		Uricase methode was used on a Dimension Vista® System (SIEMENS, Eschborn, Germany). The coefficient of variation was 1.92% at low level of control material (mean value = 291 mol/L).	visit on the question: Did you have any of the following diseases in the last 12 months? Gout or increased uric acid levels?	
**S**wiss Cohort Study on **A**ir **P**ollution **A**nd **L**ung and Heart **Di**seases in **A**dults	Prospective, population-based	1,640	asthmatics and non-asthmatics separate	28 failed genotyping, 35 low call rate (<97%), 17 non-European descent, 64 cryptic relatedness, 26 overlap with ECRHS, 12 males with high X-heterozygosity, 1 sex inconsistency, 13 missing UA levels (=1444 included in this analysis)	Two principal components were included as covariates in the regression.	Uric acid concentrations were determined by a colorimetric uricase/peroxidase method using reagents and the Modular P autoanalyser from Roche diagnostics (Rotkreuz, Switzerland). At concentrations of 203 micromol/l and 355 micromol/l the inter assay imprecision was 1% or less.	NA	Martin et al. (1997)[86], Ackermann-Liebrich et al. (2005)[87]
De Novo Replication Studies								
HYPertension in ESTonia (HYPEST)	Hypertensive cases recruited at the clinics and population-based controls [88-90]	758 of European (Estonian) ancestry	none	none	none	The venous blood for serum biomarker analysis was drawn in the morning after an overnight fast [88,90]. UA was measured by standardized assays (Cobas Integra 800Ò analytical platform, Roche Diagnostics, Inc.) at the United Laboratories, Tartu University Clinics or at the Diagnostics Division Laboratory, the North Estonia Medical Centre [90]. EURACHEM guidelines were applied to	NA	Ong et al. (2011)[88], Ong et al.(2009)[89], Juhanson et al.(2008)[90]

Nature Genetics doi:10/1038/ng.2500

						estimate measurement uncertainty (9.7%).		
KORA S2	Population-based	3,685	none	Only subjects with overall genotyping efficiencies of at least 93% were included.	none	Non-fasting blood samples were obtained from study participants. UA analyses were carried out on fresh samples. UA concentrations were measured using an uricase method (Technicon, SMAC AutoAnalyzer).	Current intake of urate-lowering medication	Wichmann et al. (2005)[47]
Ogliastra Genetic Park	Population-based study with pedigree information	9,704 of Sardinian ancestry	none	Individuals with a call rate <0.9 in de novo genotyping were excluded.	Study center was included as covariate in the regression	UA was measured in MG/DL units using TARGA 3000 with enzymatic colorimetric uricase method.	NA	Portas et al. (2010)[83], Biino et al. (2009)[84], Pistis et al. (2009)[91]
**Studies with FEUA and UUCR that are not included above**								
Hercules	Population based	374	none	Individuals with call rate below 90% were excluded. The younger of 1st/2nd degree related pairs were removed from the analysis.	First two ancestry principal components were used as covariates.	Uric acid was measured by uricase-PAP (1.0% - 0.5% maximum inter and intra-batch coefficients of variation).	NA	Bochud et al. (2009)[92]
**Incident gout studies**								
Nurses Health Study (NHS)	Gout case-control study nested within a prospective cohort (NHS)	2,275 (NHS + HPFS)	Gout cases and their matched controls	79 ids from NHS and HPFS together were removed after relatedness check, 76 ids were duplicates and 3 were removed from siblings set with high SNP missingness rate, 69 ids that did not cluster with other self-identified US whites were removed.	The top three components of genetic variation were included as covariates in the logistic and linear regressions.	UA was measured using the uricase method (Data not included as this analysis for gout).	Gout was defined by the American College of Rheumatology criteria for gout.	Choi et al. (2004)[93], Choi et al. (2004)[94], Choi et al. (2008)[95]
Health Professionals Follow-Up Study (HPFS)	Gout case-control study nested within a prospective cohort (HPFS)	2,275 (NHS + HPFS)	Gout cases and their matched controls	79 ids from NHS and HPFS together were removed after relatedness check, 76 ids were duplicates and 3 were removed from siblings set with high SNP missingness rate, 69 ids that did not cluster with	The top three principal components of genetic variation were included as covariates in the logistic and linear regressions.	UA was measured using the uricase method (Data not included as this analysis for gout).	Gout was defined by the American College of Rheumatology criteria for gout.	Choi et al. (2010)[96], Choi et al. (2010)[97]

Nature Genetics doi:10/1038/ng.2500

				other self-identified US whites were removed.				
**Study Samples of Indian Ancestry**								
London Life Sciences Population (LOLIPOP) study, LOLIPOP_IA317	Prospective, population-based	2,694	none	Duplicates, gender discrepancy, contaminated samples, relatedness, samples already in IA610	The first ten principal components were used as covariates in the regression.	Venous blood was collected into 5.0ml BD Vacutainer SST II Advance tube. Serum urate measurements were measured using the uricase method on Roche/Hitachi Cobas C 501 systems (USA).	NA	Chambers et al. (2008)[98]
London Life Sciences Population (LOLIPOP) study, LOLIPOP_IA610	Prospective, population-based	7,032	none	Duplicates, gender discrepancy, contaminated samples, relatedness	The first ten principal components were used as covariates in the regression.	Venous blood was collected into 5.0ml BD Vacutainer SST II Advance tube. Serum urate measurements were measured using the uricase method on Roche/Hitachi Cobas C 501 systems (USA).	NA	Chambers et al. (2008)[98]
London Life Sciences Population (LOLIPOP) study, LOLIPOP_IA_P	Prospective, population-based	1,005	none	Duplicates, contaminated samples, samples already in IA610 and IA317	The first ten principal components were used as covariates in the regression.	Venous blood was collected into 5.0ml BD Vacutainer SST II Advance tube. Serum urate measurements were measured using the uricase method on Roche/Hitachi Cobas C 501 systems (USA).	NA	Kooner et al. (2008)[52]
**Study Samples of African American Ancestry**								
ARIC	Population-based	2,749	not self-identified as black	Data cleaning conducted centrally at the Broad Institute	Adjustment for the first 10 principle components	Serum urate concentrations were measured with the uricase method at visit 1	Gout status was ascertained from a questionnaire at visit 4.	Iribarren et al. (1996)[6]
CARDIA	Population-based	937	none	Data cleaning conducted centrally at the Broad Institute	Adjustment for the first 10 principle components	Serum urate was measured at baseline using the uricase method.	Gout was self-reported at follow-up visits at 7, 10, and 15 years	Friedman et al. (1988)[14]
JHS	Population-based	3,443	none	Data cleaning conducted centrally at the Broad Institute	Adjustment for the first 10 principle components	Baseline serum urate was measured using the uricase method.	NA	Taylor et al. (2005)[99], Fuqua eta al. (2005)[100]
**Study Samples of Japanese Ancestry**								

Nature Genetics doi:10/1038/ng.2500

The BioBank Japan Project	Disease patients cohort [101]	15,288 of Japanese disease patients affected with each of the 21 diseases [101-103].	none	The following subjects were excluded.(i) low call rate (<98%), (ii) in 1st or 2nd kinships, (iii) outliers from East-Asian clusters in the result of principal component analysis (PCA) performed with HapMap Phase II populations, (iv) serum urate, sex, or age were not available, (v) age <18, age > 85, with dialysis treatment or with kidney failure.	Subjects who were determined to be of non-Japanese origin by self-report or by PCA were excluded. No principal component was included as covariate in the regression.	UA levels were obtained from medical records of the medical institutes which participated in the BioBank Japan Projects.	No information on the affection status of gout was obtained.	Nakamura et al. (2007)[101], Kamatani et al. (2010)[102], Okada et al. (2011)[103]
