# Supplementary material for: Decoding the mystery between hyperuricemia and atrial fibrillation: new causal links through mediating proteomics
Source: Front Endocrinol (Lausanne). 2025 May 21;16:1429465. doi: 10.3389/fendo.2025.1429465 (PMC12133553; doi:10.3389/fendo.2025.1429465)
Supplement: Supplementary file 2 [file DataSheet2.pdf]

Supplementary File 2. Extended Description of the Cohorts Used in the Atrial Fibrillation GWAS and Plasma Proteomics GWAS

Extended Description of the Cohorts Used in the Atrial Fibrillation GWAS

The HUNT Study

Sample ascertainment and phenotype definition

The Nord-Trøndelag Health Study (HUNT) is a population-based health survey conducted in the county of NordTrøndelag, Norway. Individuals were included at three different time points during approximately 20 years (HUNT1 [1984-1986], HUNT2 [1995-1997] and HUNT3 [2006-2008]).1 At each time point, the entire adult population (≥ 20 years) was invited to participate by completing questionnaires, attending clinical examinations and interviews. Participation rates have generally been high: 89.4% (n = 77,212), 69.5% (n = 65 237) and 54.1% (n = 50 807) in HUNT1, HUNT2 and HUNT3, respectively. Taken together, the health studies included information from over 120,000 different individuals from Nord-Trøndelag. Biological samples including DNA have been collected for approximately 70,000 participants. Atrial fibrillation was defined based on ICD-10 codes collected from local hospitals and out-patient clinics between 1999- 2016. Cases were defined as individual with one or more ICD-9 or ICD-10 codes specific for atrial fibrillation ("I48" or "427.3") whereas controls were all individuals without a code specific for atrial fibrillation.

Genotyping, quality control, and imputation

In total, DNA from 71,860 HUNT samples was genotyped using one of three different Illumina HumanCoreExome arrays (HumanCoreExome12 v1.0, HumanCoreExome12 v1.1 and UM HUNT Biobank v1.0). We excluded samples that failed to reach a 99% call rate, had contamination > 2.5% as estimated with BAF Regress, large chromosomal copy number variants, lower call rate of a technical duplicate pair and twins, gonosomal constellations other than XX and XY, or whose inferred sex contradicted the reported gender. Samples that passed quality control were analyzed in a second round of genotype calling following the Genome Studio quality control protocol described elsewhere. Genomic position, strand orientation and the reference allele of genotyped variants were determined by aligning their probe sequences against the human genome (Genome Reference Consortium Human genome build 37 and revised Cambridge Reference Sequence of the human mitochondrial DNA; http://genome.ucsc.edu) using BLAT. PLINK v1.90 was then used to exclude variants if their probe sequences could not be perfectly mapped, cluster separation was < 0.3, Gentrain score < 0.15, showed deviations from Hardy Weinberg equilibrium in unrelated samples of European ancestry with p-value < 0.0001), had a call rate < 99%, or another assay with higher call rate genotyped the same variant. Ancestry of all samples was inferred by projecting all genotyped samples into the space of the principal components of the Human Genome Diversity Project (HGDP) reference panel (938 unrelated individuals; downloaded from http://csg.sph.umich.edu/chaolong/LASER/), using PLINK. Recent European ancestry was defined as samples that fell into an ellipsoid spanning exclusively European populations of the HGDP panel. The different arrays were harmonized by reducing to a set of overlapping variants and excluding variants that showed frequency differences > 15% between data sets, or that were monomorphic in one and had MAF > 1% in another data set. The resulting genotype data were phased using Eagle2 v2.3. Imputation was performed on samples of recent European ancestry using Minimac3 (v2.0.1, http://genome.sph.umich.edu/wiki/Minimac3) and a merged reference panel that was constructed by combining the Haplotype Reference Consortium panel (release version 1.1) and

a local reference panel based on 2,202 wholegenome sequenced HUNT study participants. A maximal set of relatively unrelated individuals (kinship coefficient < 0.0884) was chosen using KING and FastIndep .

Association analysis

We performed testing for association with AF using a generalized mixed model including covariates birth year, sex, genotype batch, and principal components (PC) 1-4 as implemented in SAIGE. PCs were computed using PLINK. Additional filters applied to the analysis included minor allele count ≥ 10 and imputation r2 ≥ 0.3.

Michigan Genomics Initiative (MGI)

DNA from blood samples of surgical patients at the University of Michigan Health System was genotyped on a customized Illumina HumanCore Exome array. Genotypes of the Haplotype Reference Consortium were imputed into the phased MGI genotypes, resulting in dense mapping at over 20 million variants. Atrial fibrillation cases were derived from electronic health records for patients with at least 1 encounter with ICD-9 billing code 427.31 ('Atrial fibrillation'). We performed a genome-wide association analysis for Atrial fibrillation in 1,226 cases and 11,049 controls of European ancestry (excluding 1st and 2nd degree relatives). Exclusion criteria for controls included individuals with ICD-9 codes 426- 427.99. We used the Firth bias-corrected logistic likelihood ratio test with adjustment for age, sex, and principal components 1-4.

DiscovEHR Collaboration Cohort

The DiscovEHR human genetics cohort analyzed here included 58,124 consented enrollees of European ancestry from the ongoing MyCode Community Health Initiative of the Geisinger Health System ("DiscovEHR study"). Participants were recruited from outpatient primary care and specialty clinics, the cardiac catheterization laboratory, and from patient populations referred for bariatric and abdominal vascular surgery between 2007 and 2016. Clinical laboratory measurements, International Classification of Diseases, Ninth Revision (ICD-9) disease diagnosis codes, medications, and procedural codes were extracted from the electronic health records (EHR) recording a median of 15 years of clinical care.

Cases were defined as DiscovEHR participants with at least one electronic health record problem list entry or at least two diagnosis code entries for two separate clinical encounters on separate calendar days for ICD-10 I48: Atrial fibrillation and flutter. Corresponding controls were defined as individuals with no EHR diagnosis code entries (problem list or encounter codes) for ICD-10 I48.*.

Aliquots of DNA were sent to Illumina for genotyping on the Human OmniExpress Exome Beadchip. All individuals of European ancestry, as determined using principal components (PC) analysis, were imputed to the HRC Reference Panel using the Michigan Imputation Server. Markers with imputation r2 > 0.3 were carried forward for analysis with no filtering for MAF. BOLT-LMM was used to analyze BGEN dosage files, and variants were tested for association with atrial fibrillation under an additive genetic model, adjusting for sex, age, age2 , and the first four PCs of ancestry; additionally, a genetic relatedness matrix (calculated using variants with MAF > 1%, per-genotype missing data rate < 1%, and HWE P-value < 1 x 10-15) was included as a random-effects variable in the model.

The cohort details presented above are extracted directly from the original article. For further information, refer to:

Nielsen JB, Thorolfsdottir RB, Fritsche LG, et al. Biobank-driven genomic discovery yields new insight into atrial fibrillation biology. Nat Genet. 2018;50(9):1234–1239. doi:10.1038/s41588-018-

0171-3

Extended Description of the Cohorts Used in the Plasma Proteomics GWAS

The INTERVAL study comprises about 50,000 participants nested within a randomized trial of varying blood donation intervals9. Between mid-2012 and mid-2014, blood donors aged 18 years and older were recruited at 25 centres of England's National Health Service Blood and Transplant (NHSBT). All participants gave informed consent before joining the study and the National Research Ethics Service approved this study (11/EE/0538). Participants completed an online questionnaire including questions about demographic characteristics (for example, age, sex, ethnicity), anthropometry (height, weight), lifestyle (for example, alcohol and tobacco consumption) and diet. Participants were generally in good health because blood donation criteria exclude people with a history of major diseases (such as myocardial infarction, stroke, cancer, HIV, and hepatitis B or C) and those who have had recent illness or infection.

The cohort details outlined above are obtained from the original publication. For more in-depth information, see:

Sun BB, Maranville JC, Peters JE, et al. Genomic atlas of the human plasma proteome. Nature. 2018;558(7708):73–79. doi:10.1038/s41586-018-0175-2
